# The role of gender and labour status in immunosenescence of 65+ Polish population

**DOI:** 10.1007/s10522-017-9702-z

**Published:** 2017-04-25

**Authors:** Magdalena Dudkowska, Dorota Janiszewska, Anna Karpa, Katarzyna Broczek, Michal Dabrowski, Ewa Sikora

**Affiliations:** 1 Laboratory of Molecular Bases of Aging, Department of Biochemistry, Nencki Institute of Experimental Biology of Polish Academy of Sciences, Pasteur Str. 3, 02-093 Warsaw, Poland; 2 Laboratory of Bioinformatics, Nencki Institute of Experimental Biology of Polish Academy of Sciences, Pasteur Str. 3, 02-093 Warsaw, Poland; 3 Department of Geriatrics, Medical University of Warsaw, Oczki Str. 4, 02-007 Warsaw, Poland

**Keywords:** CD8+CD28−, Immune risk profile, IL8, IL10, Human ageing, T-cells

## Abstract

Women are living longer than men and it seems that one of the reasons could be better immune system of females. In Poland, contrary to many European countries, women retire earlier than men, namely at 60 and 65, respectively. We asked the question how the gender and labour status were interconnected with some immunological parameters included in the so called immune risk profile (IRP), such as CD4+/CD8+ ratio, percentage of CD8+CD28−, and NK, and the level of circulating cytokines. A total of 92 men and 100 women past the retirement age, namely 65–74 years old, still working or not, were examined. We have found statistically significant lower percentage of CD8+CD28− cells and non-statistically significant higher CD4+/CD8+ ratio in women, whereas the percentage of NK was higher in men. Moreover, the percentage of CD8+CD28− cells was negatively correlated with the CD4+/CD8+ ratio and the concentration of IL8 was positively correlated with the concentration of IL10 both in men and women. In men, the level of IL10 was higher than in women. Altogether, we found that gender, but not labour status, influences immunosenescence of the examined population of 65–74 years old Polish people.

## Introduction

Worldwide, the proportion of people aged 60 and over is growing faster than any other age group. In 2025, there will be a total of about 1.2 billion people over the age of 60. By 2050 there will be 2 billion with 80% of them living in developing countries. This will have an enormous impact on the societies. The demographic mechanisms driving this phenomenon are lower fertility rates and longer lifespans (Hayward 2001) as a consequence of the progress of civilization. Longer lifespan is usually connected with higher frequency of age-related disabilities but, on the other hand, it has been proven that ageing is an extremely plastic process, which can be accelerated, slowed down and, in some cases even stopped or reversed (Fahy et al. 2010). Human healthspan is influenced by genes (25–30%) and life conditions (70–75%) (Deelen et al. 2013), which may ensure good ageing, or in other words, active ageing, the term used by the World Health Organization Policy Framework on Active Ageing. Although the word “active” refers to continuing participation in social, economic, cultural, spiritual and civic affairs, and not just to the ability to be physically active or to participate in the labour force (Paul et al. 2012), the professional activity is a very important component of active ageing and should not be neglected. We’re living longer and we’re working longer, too. Although the study of successful ageing captures the scientists’ and public interest, the knowledge regarding the effect of lifestyle on healthy ageing, and especially the impact of the professional activity, is still incomplete. Investing in healthy ageing contributes to the labour supply and decreases the likelihood of early retirement (Vainio 2012). Although data show a remarkable gender difference—for the benefit of women—in life expectancy and mortality, including survival to an extreme age (Ostan et al. 2016), practically until this century women have retired several years earlier than men. Today, in the majority of European countries the age of retirement is equal for men and women. In Poland the regulation has been changed recently and the retirement age of 60 for women and 65 for men has been restored.

The reason of shorter life expectancy for men is still an open question, but the answer, at least partial, can be found in the differences of the immune system senescence (Berghella et al. 2012). Senescence of the immune system, commonly known as immunosenescence, is viewed often as a detrimental process. However, age-associated changes, which can be viewed as adaptive, may also had to improved function (Fulop et al. 2016). Thus, it was proposed that immunosenescence is not deterioration but rather a remodeling of the immune system (Franceschi et al. 1995) which is in agreement with our understanding of this process. Nonetheless, it seems that age-related changes of the immune system are different in men and in women (Hirokawa et al. 2013; Yan et al. 2010). The reason of this situation seems to lie in differences in sex steroid hormones (Ostan et al. 2016).

The concept of immunosenescence reflects changes in both cellular and humoral immune responses. The complex immune system remodeling observed during ageing includes also a well characterized profound modification of the cytokine network. A key feature of this phenomenon is a decrease in IL2 plasma level alongside an increase in proinflammatory molecules, such as tumour necrosis factor α (TNFα), IL6 and C-reactive protein (CRP) (Macaulay et al. 2013). These circulating inflammatory molecules are associated with a low grade inflammation that has been termed ‘inflammaging’ (Franceschi et al. 2000). It is triggered by systemic activation of both the innate and adaptive immune systems and by proinflammatory factors secreted by senescent cells that accumulate in tissues with ageing (Sikora et al. 2011). Although ageing is accompanied by a progressive increase in proinflammatory cytokines, little is known about the gender influence on their level.

In our studies we have performed a broad spectrum of analyses of social, economic and health status as well as nutrition habits in women and men of pension age, who were economically active or not. Our analysis included also some immunological parameters such as cytokine levels in serum and percentages of basal lymphocyte subpopulations and some T cells subpopulations whose changes in numbers and function are characteristic for immunosenescence. We have based our analysis on the so called “Immune Risk Profile”(IRP), which refers to a set of immunological parameters that has been used as a metric predictive of mortality of people 85 years old (Wikby et al. 1998). These parameters include inverted CD4+/CD8+ ratio due to an accumulation of CD8+CD28− T cells, poor proliferative responses to T cell mitogens, low B cell counts and seropositivity for CMV (Fulop et al. 2013). Although the IRP was defined as a metric predictive of 2, 4 and 6 year mortality in people 85 years old at baseline we hypothesized, when assessed in a cohort of younger people (65+), these immunological features may contribute to our understanding the cellular and molecular mechanisms underlying the gender and labour influence on the condition of their immunosystem.

In this paper we show only the “immunological part” of our studies, which indicate for the existence of some differences between women and men, which could be a contributory factor to women living longer than men. Interestingly, the labour status appears to have a slight influence on the immunological parameters in elderly women but not in men.

## Methods

### Subjects

Healthy male and female volunteers, aged from 65 to 74 years, retired at least for 3 years or still working at least half time, were selected on the basis of detailed interview. Standardized questionnaire contained questions about the health status and medications, among others. Individuals with various health problems such as dementia, diabetes, autoimmune diseases, acute or chronic infectious disease, those being on anticancer treatment or on medication with a known influence on immunological factors (e.g., corticosteroids) were excluded from the study. A total of 92 men and 100 women were examined. Routine laboratory examinations of these individuals were performed to assess the complete cell count, CRP and glucose. Table 1 shows the numbers of male and female subjects and their age.

**Table 1:** Number of female and male subjects

Age (years)	65–69	70–74	Total
Female			
Working	34	17	100
Non-working	27	22	
Male			
Working	35	10	92
Non-working	21	26	

### Ethical approval

This study was conducted in compliance with the Declaration of Helsinki and applicable national laws and regulations, and it was approved as KB 203/2014 by the Ethics Committee of the Warsaw Medical University. Written informed consent was obtained from all subjects.

### Blood specimens

Fifteen milliliters of blood was collected: 8 ml for the routine laboratory examinations (complete cell count, CRP and glucose) and 2 ml in a tube containing K_2_EDTA (BD Vacutainer, 368841) for lymphocyte phenotyping and 5 ml in a tube with clot activator and gel for serum separation (BD Vacutainer, 367955) for cytokine measurement.

### Cytokine measurement

Separated serum was centrifuged twice: at 1500×*g* for 10 min, then at 3000×*g* for 10 min. Obtained supernatant was frozen at −80 and kept until cytokine measurement. Cytokines- IL1β, IL6, IL8, IL10, IL12, TNFα were estimated using BD LSRFortessa flow cytometer, equipped with 488 and 640 nm lasers and BD FACSDiva 6.2 software. Bead fluorescence was detected with 575/26, 670/14 and 780/60 band passes (BPs) and gated according to Cytometric Bead Array (CBA) Human Enhanced Sensitivity Master Buffer Kit manual. Data were analysed with BD FCAP Array TM 3.0 software.

### Cell phenotyping

We used the BD Multitest™ IMK kit (340503) to identify and determine the percentage and absolute counts of the following mature human lymphocyte subsets in erythrocyte-lysed whole blood: T lymphocytes (CD3+), B lymphocytes (CD19+), T lymphocytes (CD3+CD4+ and, CD3+CD8+) and natural killer (NK) lymphocytes (CD3−CD16+ and/or CD56+). To define single subsets of CD28+ and CD28− among CD8+ cells we used BD Simultest CD8CD28 (340031). Samples were recorded and analysed on the FACS Calibur (Beckton-Dickinson, Warsaw, Poland) using the Cell-Quest software (BD) and Multiset software (Beckton-Dickinson), respectively.

### Statistical analysis

In our study, we had two types of variables: the explaining variables, used to group our subjects, namely: gender, labour status, and the age category and quantitative dependent variables, namely: the percentage of CD8+CD28− and NK cells, the CD4+/CD8+ ratio and the concentrations of six circulating cytokines (fg/ml), of which two (IL8 and IL10) are shown. The Kolmogorov–Smirnov test demonstrated that the dependent variables did not follow the normal distribution, therefore non-parametric tests were used. The data were imported into the R statistical software environment and analysed using the Wilcoxon rank sum test (pairwise comparisons) or Kruskal–Wallis Chi squared test (comparison among multiple groups). The Spearman’s rank-order correlation coefficient ρ was used to measure the correlation between different immunological parameters of the same subject.

## Results

The purpose of this study is to analyse gender and labour status influence on the percentage of CD8+CD28− and NK cells of peripheral blood lymphocytes and on the CD4+/CD8+ ratio and serum cytokines in blood obtained from 65 to 74 years old healthy male and female volunteers.

### Interconnections between gender and immunological parameters

**Fig. 1:**
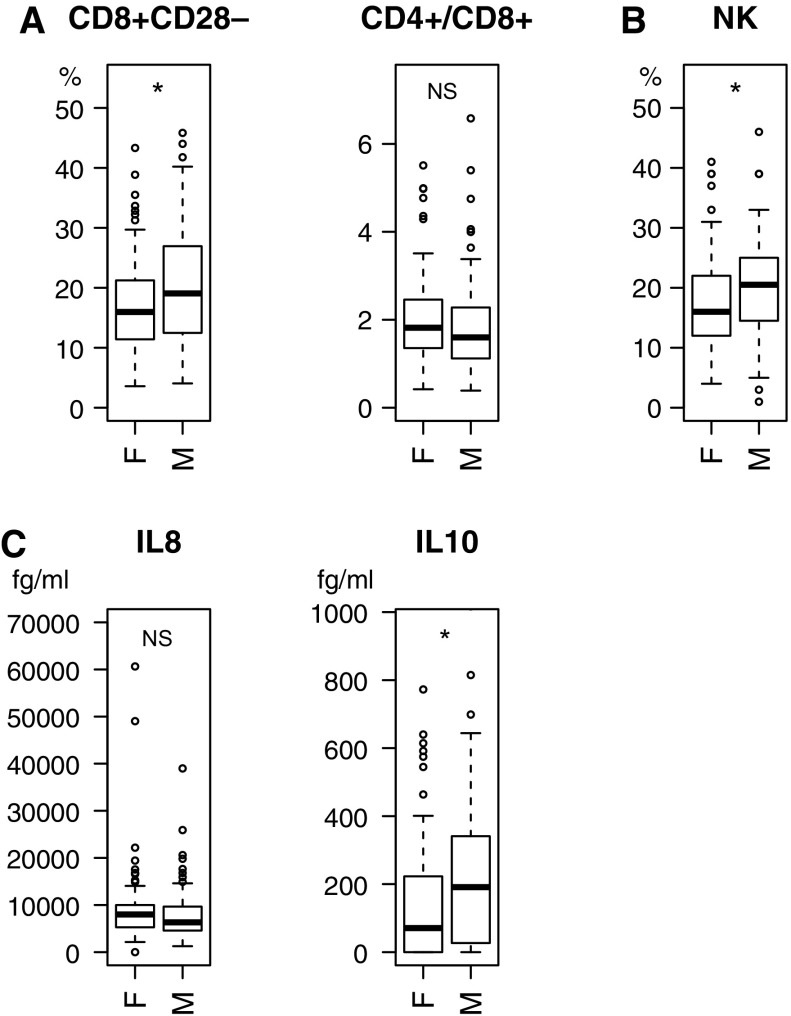
Distribution of values of immunological parameters: percentage of CD8+CD28− T-cell subpopulation of lymphocytes and the ratio of CD4+/CD8+ lymphocytes (**a**), percentage of natural killers NK cells (**b**), IL8 and IL10 serum concentration (**c**) in the elderly subjects grouped by gender (*F* female, *M* male) are shown as box-plots with default options. For IL10 and IL8 the *y* axis range was trimmed to the range shown. The observations outside this range were thus excluded from the plot, but they were included in statistical analysis. The *box* marks the quantiles 25, 50 (median) and 75%. The *whiskers* extend to the most extreme data point, that is no more than 1.5 times the interquartile range for the *box*. Values outside this range are shown as *dots*. Statistical significance: * for p < 0.05; *NS* not significant

**Fig. 2:**
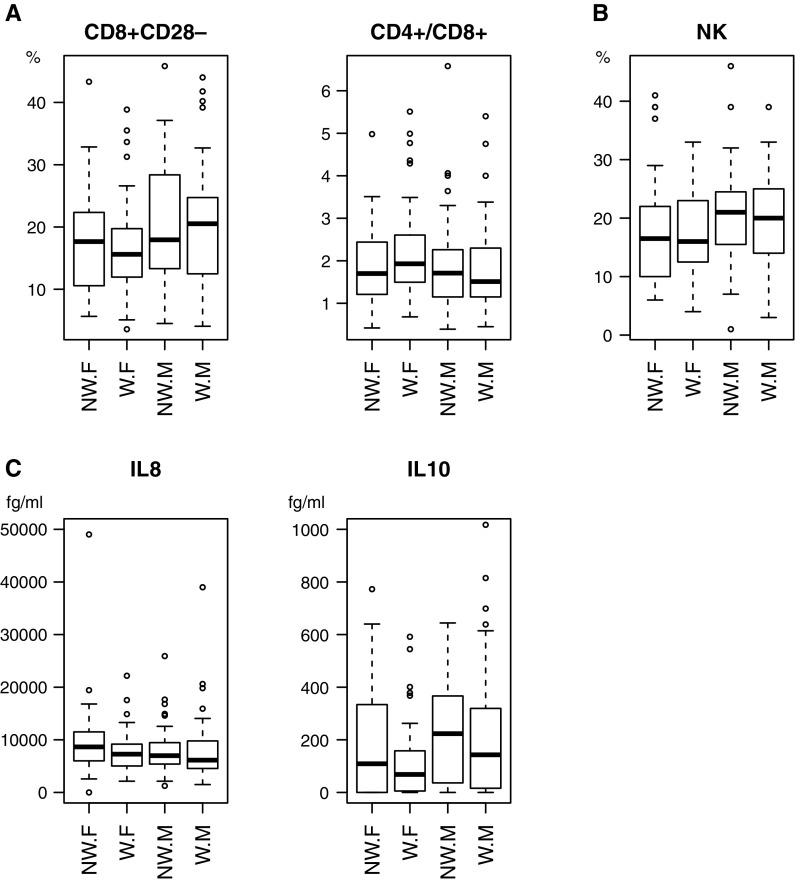
Distribution of values of immunological parameters: percentage of CD8+CD28− T-cell subpopulation of lymphocytes and the ratio of CD4+/CD8+ lymphocytes (**a**), percentage of natural killers NK cells (**b**), IL8 and IL10 serum concentration (**c**) in the elderly subjects grouped by gender (*F* female, *M* male) and labour status (*W* working, *NW* not working), are shown as box-plots with default options. For IL10 and IL8 the *y* axis range was trimmed to the range shown. The observations outside this range were thus excluded from the plot, but they were included in statistical analysis. The *box* marks the quantiles 25, 50 (median) and 75%. The *whiskers* extend to the most extreme data point, which is no more than 1.5 times the interquartile range for the *box*

Figures 1 and 2 show distribution of values of the percentage of CD8+CD28− T-cells and NK cells in blood lymphocytes, the CD4+/CD8+ ratio and the concentration of serum IL8, IL10 cytokines in the studied subjects grouped by the gender only (Fig. 1) or gender and the labour status (Fig. 2).

We observed higher amount of CD8+CD28− T cells (W = 3620, p = 0.014, Wilcoxon rank sum test) in males than in females in this age group (Fig. 1a). Moreover, it seems that the CD4+/CD8+ ratio was lower in men than in women of the same age although this difference was not statistically significant (W = 5221.5, p = 0.081, Wilcoxon rank sum test).

**Fig. 3:**
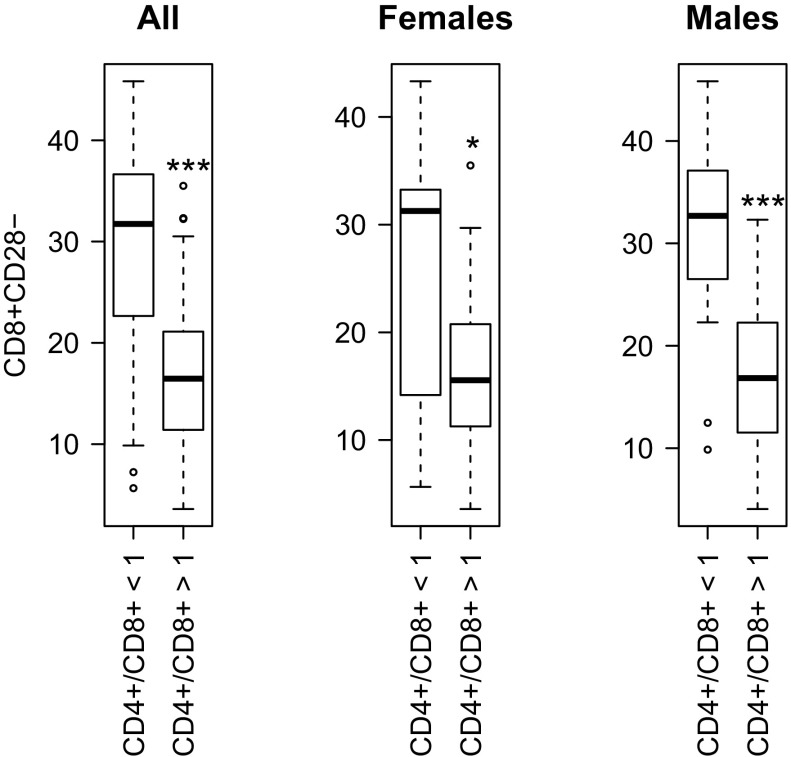
Percentage of CD8+CD28− T-cell subpopulation of lymphocytes in groups with the ratio of CD4+/CD8+ lower or above 1 in all elderly subjects and grouped by gender. Statistical significance: * for p < 0.05; *** for p < 0.001

Moreover, as a reverse CD4+/CD8+ ratio ≤1 was postulated for IRP (Wikby et al. 1998), we analyzed percentage of CD8+CD28− cells in subjects with such a ratio. Interestingly, among 100 female subjects exactly 10% were characterized by CD4+/CD8+ ≤1, while for 92 males this figure was 22.8%. The median of the percentage of CD8+CD28− T cells for both sexes in this selected group accounted for slightly above 30%, which is almost two times higher than for the entire examined population (17.44%). Additionally, for both sexes (Fig. 3), the percentage of CD8+CD28− cells was lower in subjects with the CD4+/CD8+ ratio above one than in the subjects with this ratio below one. The effect was strong and highly significant (Mann–Whitney U test p value = 7.6 × 10^−9^). The size of the effect (two fold difference in medians) was the same in both sexes (Fig. 3, females vs males) but due to higher variability in females its significance was greater among males (p values: 1.4 × 10^−7^ and 0.03; respectively).

Figure 1b shows the percentage of NK cells of peripheral blood lymphocytes. From our data it emerges that the median of percentage of NK cells for women is lower than for men (Fig. 1b). Indeed, the statistical analysis of NK level revealed significant difference in subgroups divided due to gender (W = 3660, p = 0.019, Wilcoxon rank sum test). Comparative analysis of these subgroups showed that males had significantly higher NK level (M = 20.07; SD = 8.15) than females (M = 17.73; SD = 8.02), thus again indicating the difference between 65 and 74 year old men and women.

We have analysed six cytokines in the serum of the examined women and men: IL1β, IL6, IL8, IL10, IL12p70 and TNFα. However, only IL8 and IL10 were detected in the sera of almost 100 and 75% of subjects, respectively. Figure 1c shows their level. IL1β, IL6 and TNFα were detected only in 11% whereas IL12p70 only in 2% of subjects. Our results (Fig. 1c) indicate a higher level of IL10 in men than women (W = 3657, p = 0.018, Wilcoxon rank sum test).

### Interconnections between gender and labour status variables with immunological parameters

To explore possible dependencies between the variables, we decided to group the dependent variables by gender and labour status (Fig. 2). For CD8+CD28− the difference in the medians due to gender was greater among working subjects. This modifying effect was, however, not significant (Kruskal–Wallis χ^2^ = 6.411, p = 0.093). Within each gender group, the working subjects had lower median IL10 concentrations than the non-working subjects but this modifying effect was not significant (Kruskal–Wallis χ^2^ = 6.493, df = 3, p = 0.090).

### Correlations between the immunological parameters

**Fig. 4:**
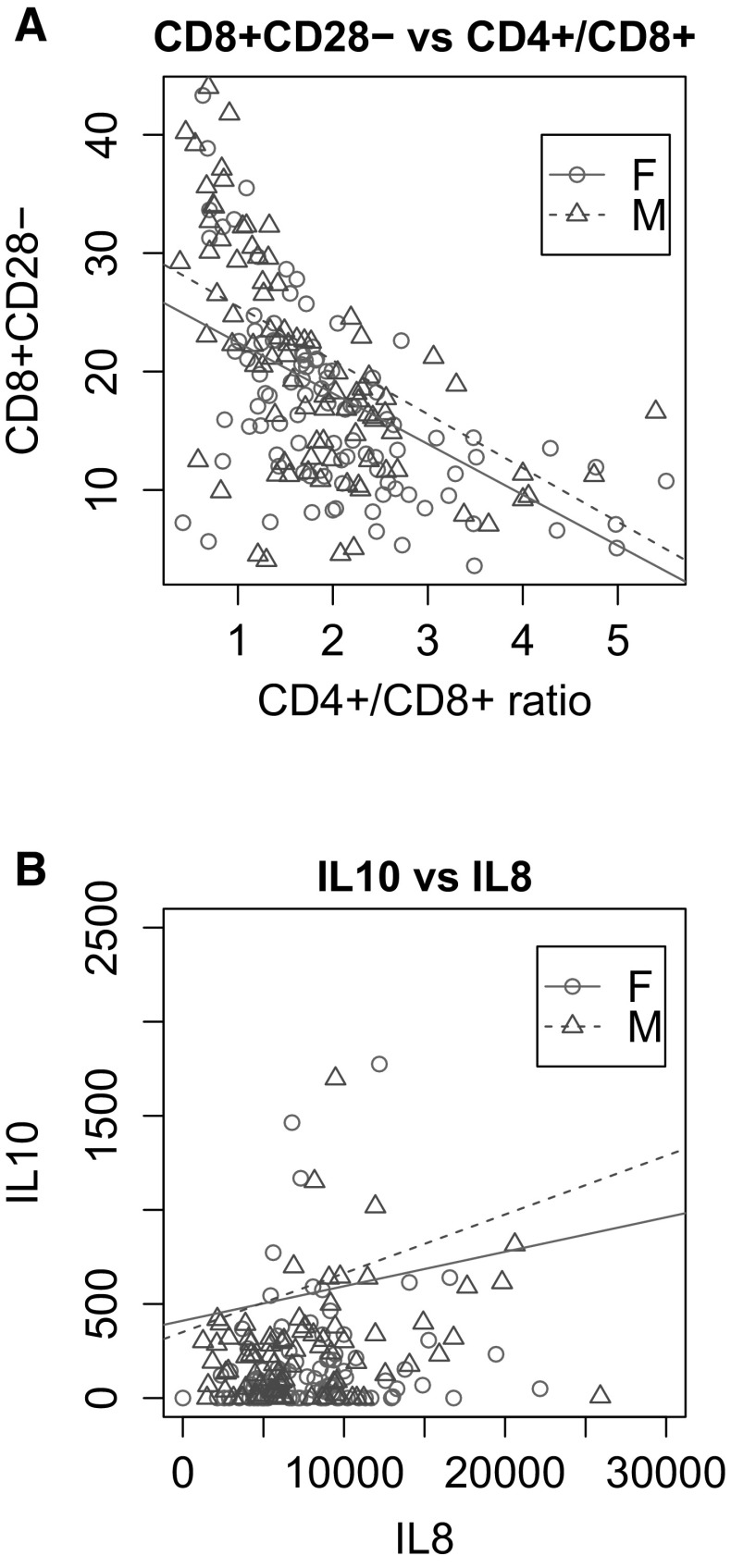
Pairwise plot of the values of two immunological parameters: percentage of CD8+CD28− T-cell subpopulation of lymphocytes and the ratio of CD4+/CD8+ lymphocytes (**a**), IL8 and IL10 serum concentration (**b**) in the elderly subjects, (female—*red circle*, male—*blue triangle*). (Color figure online)

Pairwise plot (Fig. 4a) demonstrates the presence of a strong inverse relationship between two important immunological parameters: CD8+CD28− and CD4+/CD8+ from the same subjects. This is also confirmed by their very strong negative correlation, with ρ = −0.628 (Spearman’s rank-order correlation).

A similar pairwise plot for IL8 and IL10 is shown in Fig. 4b. Detailed analysis of correlations in selected subgroups was also performed and is presented in Table 2.

**Table 2:** Summary of correlation values for the variables analyzed in selected subgroups

Group variable	Levels (gv)		CD8+CD28−	
			ρ	p
Sex	Male (M)	CD4+/CD8+	−0.677	<0.001
	Female (F)	CD4+/CD8+	−0.581	<0.001
Labour status	M—working	CD4+/CD8+	−0.611	<0.001
	F—working	CD4+/CD8+	−0.645	<0.001
	M—retaired	CD4+/CD8+	−0.740	<0.001
	F—retaired	CD4+/CD8+	−0.467	<0.001
Age	M: 65–69	CD4+/CD8+	−0.672	<0.001
	F: 65–69	CD4+/CD8+	−0.511	<0.001
	M: 70–74	CD4+/CD8+	−0.635	<0.001
	F: 70–74	CD4+/CD8+	−0.727	<0.001

Group variable	Levels (gv)		IL10	
			ρ	p
Sex	M	IL8	0.310	<0.01
	F	IL8	0.214	<0.05
Labour status	M—working	IL8	0.401	<0.01
	F—working	IL8	0.021	0.883
	M—retaired	IL8	0.204	0.168
	F—retaired	IL8	0.329	<0.05
Age	M: 65–69	IL8	0.299	<0.05
	F: 65–69	IL8	0.093	0.485
	M: 70–74	IL8	0.281	0.097
	F: 70–74	IL8	0.386	<0.05

In subgroups divided according to gender the correlations between the number of CD8+CD28− cells and the CD4+/CD8+ ratio were statistically significant and strong in each subset. In both groups the correlation was negative among both men and women, with ρ = −0.677 and −0.581, respectively (Fig. 4a).

In subgroups distinguished according to labour status and gender the correlation between the CD8+CD28− level and the CD4+/CD8+ ratio was statistically significant and negative in each subset. In the case of three subgroups we found strong correlations: working men ρ = −0.611; working women ρ = −0.645; not working women, ρ = −0.467. In the case of the group of not working men the correlation was very strong ρ = −0.740.

In subgroups divided according to age and gender the correlation between CD8+CD28− level and the CD4+/CD8+ ratio was negative and statistically significant. In the case of three subgroups we found strong correlations: men aged 65–69 ρ = −0.672; female aged 65–69 ρ = −0.511; male aged 70–74 ρ = −0.635. In the case of the group of women aged 70–74 the correlation was very strong ρ = −0.727.

Our results indicate that both parameters are strictly connected. Namely, the more CD8+ cells the higer percentage of cells lacking the CD28 co-receptor.

Moreover, in subgroups based on gender, correlation between the concentration of IL8 and IL10 was positive and statistically significant. In the case of both subgroups we found moderate correlations: men, ρ = 0.310; women, ρ = 0.214.

In subgroups distinguished according to professional activity and gender the correlation between IL8 and IL10 concentration was substantial and positive in two sub-groups (working men and women). In the case of both subgroups we found moderate correlations: active men, ρ = 0.401; active women, ρ = 0.329.

In subgroups divided according to age and gender this correlation was relevant and positive in two subsets (males 65−69 and females 70−74), with ρ = 0.299 and 0.386, respectively.

## Discussion

Accumulating evidence has shown that age-related immunological decline occurs primarily in T cell-dependent immune functions and is mainly caused by thymic involution that begins in the early phase of life. Among blood cells, the T-cell pool undergoes a striking age associated remodelling, exhibiting an inverted CD4+/CD8+ T-cell ratio alongside a diminution in naïve T cells and accumulation of more differentiated memory cells, most abundantly observed within the CD8+ T-cell compartment (Pawelec et al. 2009). It is known that T-cell activation leads to CD28 down-regulation. Indeed, various models of T-cell replicative senescence show that subsequent rounds of cell divisions eventually lead to accumulation of CD28-negative cells, which are the progeny of CD28-positive ones. Also, in vivo, the accumulation with age of CD8+CD28− and, to a lesser extent CD4+CD28− cells is observed (reviewed by Sikora 2015). Indeed, profound differences in the number of CD8+CD28+ cells in people of different age with the lowest amount in centenarians was observed (Brzezinska 2005). Along with the age-associated increase in CD8+CD28− subpopulation a drop in the CD4+/CD8+ ratio was observed in that study (Brzezinska 2005).

The phenotypic changes occurring during senescence were included in the so called immune risk phenotype (IRP), a cluster of immune parameters associated with poor immune function and predictive of earlier mortality. IRP is characterized by an inverted CD4+/CD8+ ratio, increased levels of CD8+CD28− T cells, poor T-cell mitogen responses, low B-cell counts (Wikby et al. 1998) and cytomegalovirus (CMV) seropositivity (Pawelec et al. 2014). Although IRP was originally identified as predictive of an increased 2-year mortality in people 86–94 years old subsequent studies reveled that IRP could characterize also the population of 6-years old people (Strindhall et al. 2013). Accordingly, we used this index for comparison of the condition of the immune system in 65−74 years old females and males. We have found statistically higher amount of CD8+CD28− in males than in females in this age group. It appeared that the lower CD4+/CD8+ ratio in men was not statistically significant, however, interestingly, two times more males (over 20% from all of them) than females (10%) have the CD4+/CD8+ ratio equal or lower than 1. Similarly, the proportion of 66 years old individuals with an inverted CD4+/CD8+ ratio was significantly higher in men than in women (Strindhall et al. 2013). In our studies we did not check CMV seropositivity, which has been shown to be strictly connected with IRP (Pawelec 2014) but we think it rather unlikely that more men than women were CMV infected. Nevertheless, we feel confident to conclude that IRP profile can be used to describe differences in immunosenescence rate between the male and female subjects included in our study.

It has been reported that with ageing in humans there is an increase in the percentage of NK cells and decrease in their functionality (Facchini et al. 1987), but the NK cell subsets are differentially affected (Gayoso et al. 2011). To our knowledge there is no data describing gender differences of NK number and functionality. We have found a significantly lower NK percentage in women than men. This again indicates that the senescence of the immune system in man may proceed along a different trajectory than in women.

All the cellular components of the immune system are orchestrated by cytokines that play a very important role in modulating immune cell functions because they build signal pathways that facilitate communication between different cells. It was shown that ageing is characterized by an increased serum level of a variety of pro-inflammatory cytokines (IL6, IL15, IL8) (Franceschi et al. 2007). In our studies a detectable level of IL8 and IL10 was found in majority of the examined subjects.

The family of chemoattractant cytokines (e.g., IL8), called chemokines, has a key function in host defense since they act in the recruitment and activation of leukocytes at the site of infection (Mukaida et al. 1998). Increase secretion of these cytokines is a components of the so called senescence associated secretory phenotype (SASP) characteristic for senescence cells (Coppe et al. 2008). IL10, in contrast to IL8, is an anti-inflammatory cytokine (Saraiva and O’Garra 2010) and it was shown to increase in elderly women, but not men (Ostan et al. 2016). Others found the presence of a −1082GG genotype variant in *IL10* gene promoter, suggested to be associated with high IL10 production, in Italian male centenarians. The authors claim that it significantly increases the probability to reach the extreme limit of human lifespan in men (Lio et al. 2002). However, it cannot be excluded that high IL10 production is a general future of male immune system as it was shown that testosterone increases IL10 synthesis (Klein et al. 2010). On the other hand, it was shown that IL10 level increased in aged women but not in men (Ostan et al. 2016) and increase of this cytokine was observed in postmenopausal women (Deguchi et al. 2001). From our study it emerges that men at age 65–74 have a higher median amount of IL10 in serum than women of the same age. This is an interesting observation but, based on our studies, we cannot conclude whether this difference appeared with ageing or it is just gender characteristics. Considering studies performed by Bartlett and others we can argue against CMV infection as cause of these differences (Bartlett et al. 2012).

## Conclusions

Professional activity is a very important part of environment which, as we know, influences the longevity in about 70% (Deelen et al. 2013). Thus, we could expect to see its influence on the measured immunological parameters. However, it is of note that all subjects examined in our study were working until their retirement age and the discrimination between working and not working ones covers only a short period of their life.

Women are living longer than men. There are also some data showing that immunosenescence is faster in males than females. Our results based on measurements in the elderly (aged 65−74 years) of some canonical immunological parameters known to change with age, namely CD8+CD28− and NK numbers, CD4+/CD8+ ratio and serum cytokine levels, point, with the exception of cytokine levels, to rather faster ageing of the immune system in men than women. Interestingly, the higher level of IL10 in men than in women can argue for some positive differences in low inflammaging status in favour of men. As both sex groups consisted of people who were or were not professionally active at least 3 years after the age of retirement, which in Poland is 60 years for women and 65 years for men, we were interested how the labour status after the retirement age is interconnected with immunosenescence. It appeared that women that were still working could be in a slightly, but not statistically significant, better condition than those not working. We think that, at least when immunosenescence is considered there is no reason for an earlier retirement of women than men.
